# A New Source of Revenue for the Enterprising Medical Publisher

**Published:** 1888-08

**Authors:** 


### A NEW SOURCE OF REVENUE FOR THE ENTERPRISING MEDICAL PUBLISHER.

Surgeon General Hamilton, of the Marine Hospital Service, U. S. A., has been a subscriber to DANIEL'S TEXAS MEDICAL JOURNAL from the beginning to the present time. To secure the subscription of any of the departments of the United States Government, it is necessary to enter upon a schedule submitted, the price at which it is proposed to furnish the journal, for a stated time; as in the case of any other supplies. This is understood to be a mere matter of form. Accordingly, for the past three years, at the beginning of each volume (July), we have had submitted to us a schedule of "proposals for supplies," and opposite the item, "DANIEL'S TEXAS MEDICAL JOURNAL one year," we have inserted the subscription price, "$2," and returned the same, receiving, generally, by return mail, a Treasury draft for the amount. In July, here came the "proposal for supplies," and in the list of publications enumerated, was "DANIEL'S TEXAS MEDICAL JOURNAL, one copy." As usual, the price, $2, was inserted opposite it, and the document returned.

In due time we received an official letter from the Treasury Department Marine Hospital Service, in which we confidently expected to find the customary $2 Treasury draft. Imagine our surprise and disgust, on opening the document, to find, instead of our $2, official information that our "proposal to furnish one copy DANIEL'S TEXAS MEDICAL JOURNAL was not accepted, Messers. Cupples & Hurd, of Boston, having offered to furnish it for less." [ ! ! ! ! ! ! ! ]

Cupples & Hurd are medical publishers, and issue, amongst other periodicals, the Boston Medical and Surgical Journal. They have been great sticklers for etiquette and ethics; and our readers may remember a letter in this JOURNAL, some months ago, written them by Mr. Champney, President of the Bush Manufacturing Company, proprietors of Bovinine, scoring them for a squeamishness which objected to the publication in their pure, blue-blood journal anything so sanguinary, plebeian and unethical as "Bovinine." We had not been exchanging with the Boston Medical and Surgical Journal prior to the publication of that letter; but (we suppose) it tickled them so they requested to be placed upon our exchange list.

It must have occurred to these enterprising gentlemen that, securing our JOURNAL free, they could turn an honest penny by selling it, second hand, to Uncle Sam at a reduced price, for we cannot imagine how else they expected to get it; and accordingly, when the schedules alluded to were sent out from the Surgeon General's office, these e. g., (we presume), filled out the entire list. We do not know at what price they offered to sell *us* to the Surgeon General, but whatever it was, it was less than *two dollars*, our regular subscription price, and—*they got the contract!* Thus we lost a subscriber! How is that for ethics and etiquette, and professional courtesy?

Here is enterprise for you. Something new in medical journalism,—exchanges turned to pecuniary profit, and sold at a discount. Here is a trick that should put to shame the originator of the wooden nutmeg racket. (He was a blue-blood, we believe, from "Bosting.") We are like the fellow who didn't swear when he was cheated, because he "couldn't do justice to the subject." We have no words with which to express our "feelinks." The pusillanimousness of the transaction; the infinitesimal *smallness* of such a deal on the part of a large and well-known medical publishing house as to sell, second hand (we presume), to the United States Government, an exchange journal, for about a dollar and a half, is so stupendous in its littleness as to be paralyzing to one's thinking apparatus. It double discounts the antiquated yarn of the fellow who stole the sweetening out of the negro baby's ginger cake; and so we wrote Messrs. C. & H.

In course of mail—after they had about had time to get our letter, we received from them a postal card, saying, "Send one copy of your JOURNAL to Surgeon General, M. H. S., Washington, and send us bill at *best discount.*" We billed them at $2, the price the Surg. General had paid us for the past three years, and wrote Dr. Hamilton, that as we would take steps to frustrate the little scheme of the Boston bluebloods--putting it out of their power to fulfill their contract—by stopping our exchange, we would, rather than that the Treasury Department, the M. H. S., and the sick and disabled seaman and officers should be deprived of the light and comfort of the Red Back "Star of the West," put the M. H. Service on our *complimentary* list. Messrs. Cupples & Hurd cannot well refuse to pay for the JOURNAL—at $2,—and if they do, they have made a nice little spec. by which they must charge to profit and loss about fifty cents a year. Big thing that.

Let our contemporaries look to their exchanges. C. & H. presumably sell them to the exchange's own subscribers *at less than the publishers themselves can do.* If this game has been attempted on a scale large enough to make it pay, (as we apprehend it has,)—if they, C. & H., bid on all the publications enumerated on Dr. Hamilton's schedule, and as in our case, *underbid the publihers*, good-by-John, to their "exchange list." This is nearer selling a birthright than anything since the days of Esau. *Vive la* Yankee! Wooden nutmegs, *redivivus.* Bosting still ahead in "culcher" and Yankee shrewdness.

Since the above was in type we have received a letter from Cupples & Hurd, in reply to ours of the 11th. They *refuse to pay the subscription*—on the ground that "now [we] you have placed the Surgeon General on your [our] free list, there is no use in sending duplicate copies"!! They also say they did not expect to make profit on any one item, but on the *entire list* of publications furnished the Surgeon General." . . . [We told you so.] They claim that they expected *us* to send the JOURNAL and they would pay for it "at our best discount." Just why they should inserfere with one of our regular paying subscribers and propose to sell him *our* JOURNAL *at a discount*, when he was paying full rate—expecting themselves a further discount—is one of those things "which no fellow can find out." Had they secured us a new subscriber at full rate and sent us the name, we would gladly have allowed them the usual agent's commission. *Too thin.* There is no other construction to be put upon it than that they expected a profit; and no how else could they get it than by sending their own copy, second hand. If they had *paid the bill*,—which they now shirk, on a subterfuge, it would have been *some* evidence of good faith. A poor "get out," Messrs. C. & H.

